# Age Associated With Preference for More and Emotionally Meaningful Information in Time-Use Decisions

**DOI:** 10.1093/geroni/igaa057.1621

**Published:** 2020-12-16

**Authors:** Yochai Shavit, Laura Carstensen

**Affiliations:** Stanford University, Stanford, California, United States

## Abstract

Socioemotional selectivity theory posits that goals change as a function of perceived time-horizons. As people age and time-horizons grow increasingly limited, people prioritize goals realized in the present over goals that will be realized in the distant future. Because goals that are emotionally meaningful are realized in their pursuit SST predicts that such goals will be prioritized by older people. Previous investigators have observed that age is negatively associated with search for information in decision making and attributed the finding to an effort to reduce cognitive load. In the present study, we hypothesized that because time becomes increasingly valued as it grows scarce, decisions about time use will show different patterns. We further hypothesized that age is associated with preference for information about present-oriented, namely emotional, benefits of activities over future-oriented benefits. 262 participants, aged 18-93 years, were presented with a hypothetical scenario inviting them to participate in one of four activities. On a grid containing 12 cells, participants were given the option to review information about activities’ emotionally meaningful and future-oriented benefits. Participants could review as many cells as they wished before indicating their interest in the activity. Across all activities, age was associated with preference for reviewing emotionally meaningful information. Moreover, in this scenario age was associated with reviewing more not less information. Age-related preferences for more information was apparent despite a negative age association with recall-memory, suggesting that cognitive load does not deter information search when decisions align with socioemotional goals.

